# Advances in NLRP3 inflammasome research in viral infections: evolution, trends, and future directions

**DOI:** 10.3389/fcimb.2026.1956027

**Published:** 2026-09-10

**Authors:** Fan Wu, Feng Xiong, Shuang Jin, Xuefei Hu, Lei Zhang, Sicheng Li

**Affiliations:** 1 Institute of Acupuncture and Moxibustion, Jianghan University, Wuhan, China; 2 College of Traditional Chinese Medicine, Department of Medicine, Jianghan University, Wuhan, China; 3 Department of Surgery, Yanqing Hospital of Beijing Chinese Medicine Hospital, Beijing, China; 4 Department of Emergency, Yanqing Hospital of Beijing Chinese Medicine Hospital, Beijing, China; 5 Department of Rehabilitation, Hubei Provincial Hospital of Integrated Chinese and Western Medicine, Wuhan, China

**Keywords:** bibliometric analysis, innate immunity, NLRP3 inflammasome, pyroptosis, research trends, viral infection

## Abstract

**Objectives:**

The NLRP3 inflammasome is a central innate immune sensor in virus-associated inflammation, yet the global research landscape of NLRP3 in viral infections remains unmapped. This study aimed to characterize its structure, evolution, research hotspots, and translational status.

**Methods:**

We performed a dual-database bibliometric analysis of publications from the Web of Science Core Collection (WoSCC) and Scopus through April 28, 2026, analyzing each database independently with cross-validation. Publication trends, collaboration networks, journal and co-citation patterns, citation bursts, keyword co-occurrence, and trend topics were examined using bibliometrix, VOSviewer, and CiteSpace.

**Results:**

Publications from WoSCC (n = 1,544) and Scopus (n = 1,495) were analyzed independently and cross-validated. Annual output rose steadily from 2009 to a peak in 2025, expanding most steeply during 2019–2022. China and the United States dominated publication output, and cumulative all-affiliation output suggests that China overtook the United States around 2021–2022 despite its lower international collaboration ratio. The most productive institutions, led by the Chinese Academy of Sciences, shifted toward Chinese affiliations after 2018–2020. High-volume journals such as Frontiers in Immunology sustained routine output, whereas a small number of landmark papers in Nature dominated citation influence. Three recurrent hotspots emerged: viral triggers and NLRP3 activation mechanisms, the balance between antiviral defense and hyperinflammation, and NLRP3-associated cell death with therapeutic targeting. After 2022, trend topics shifted toward therapy, NRF2 regulation, diagnosis, and brain pathology, while clinical translation remained largely preclinical.

**Conclusion:**

This study delineates the advancements in NLRP3 inflammasome research related to viral infections by systematically mapping its evolutionary trajectory, core research trends, and priority future directions. The analysis outlines the structural and collaborative landscape of the field and may inform future mechanistic investigations, cross-institutional collaborations, and clinical research.

## 1 Introduction

Viral pathogens impose a persistent global public health burden, with influenza A virus, severe acute respiratory syndrome coronavirus 2 (SARS-CoV-2), respiratory syncytial virus (RSV), hepatitis viruses and herpesviruses triggering recurrent seasonal epidemics and cross-border pandemics (World Health Organization, 2024). A systematic clinical meta-analysis has documented overlapping severe manifestations and elevated mortality risk among patients coinfected with influenza and SARS-CoV-2 (Varshney et al., 2023). Clinical and translational evidence indicates that life-threatening complications such as acute respiratory distress syndrome (ARDS), multi-organ failure, and lethal pulmonary injury are driven less by direct viral cytotoxicity than by dysregulated host innate inflammatory cascades, often described as a cytokine storm (Fajgenbaum and June, 2020; Huang et al., 2020); this dysregulation remains a major barrier to antiviral and anti-inflammatory therapy. Identifying the core innate immune hubs that govern viral hyperinflammation is therefore essential for developing host-directed antiviral therapies.

The innate immune system serves as the frontline antiviral defense via pattern recognition receptors (PRRs), which sense pathogen-associated molecular patterns (PAMPs) and damage-associated molecular patterns (DAMPs) released by injured host cells (Kumar et al., 2011). Among the characterized inflammasome complexes, the NOD-like receptor family pyrin domain-containing protein 3 (NLRP3) inflammasome responds to an unusually broad range of viral and cellular stress cues and has been implicated as a central driver of virus-associated inflammatory disorders across multiple tissues (Chen et al., 2023). The canonical NLRP3 complex consists of three core components: the sensor NLRP3, the adaptor apoptosis-associated speck-like protein containing a caspase recruitment domain (ASC; encoded by the PYD and CARD domain-containing protein gene, PYCARD), and the effector pro-caspase-1 (Schroder and Tschopp, 2010; Broz and Dixit, 2016). Upon viral stimulation, NLRP3 undergoes oligomerization to recruit ASC and pro-caspase-1, inducing caspase-1 self-cleavage and subsequent maturation of pro-interleukin-1β (pro-IL-1β) and pro-interleukin-18 (pro-IL-18), together with cleavage of gasdermin D (GSDMD) (Man et al., 2017). GSDMD-mediated permeabilization of mitochondrial membranes can further amplify inflammatory cell death cascades during viral infection (Miao et al., 2023). One well-characterized viral trigger of NLRP3 assembly is influenza A virus, which disrupts trans-Golgi network architecture to promote inflammasome aggregation (Pandey and Zhou, 2022).

Landmark mouse studies showed that NLRP3 contributes to *in vivo* antiviral defense against influenza A virus, providing the experimental framework for much subsequent viral inflammasome research (Allen et al., 2009; Thomas et al., 2009). Follow-up influenza-focused mechanistic work suggested a dual character of NLRP3 signaling: moderate inflammasome activation may facilitate viral clearance, whereas sustained overactivation can aggravate tissue inflammatory damage (Sarvestani and McAuley, 2017). The coronavirus disease 2019 (COVID-19) pandemic substantially accelerated research on NLRP3-mediated respiratory inflammation. Preclinical models indicated that the SARS-CoV-2 nucleocapsid protein can promote NLRP3 inflammasome activation (Pan et al., 2021), and patient-level studies reported that inflammasome activation and circulating cytokine signatures, including elevated IL-1β and IL-18, are associated with COVID-19 severity, intensive care unit (ICU) admission and mortality (Del Valle et al., 2020; Rodrigues et al., 2021). Comprehensive reviews have summarized the multi-layered upstream signaling pathways controlling NLRP3 priming and activation across various viral pathogens (Yue et al., 2024; Shin et al., 2026).

Over the past two decades, research on NLRP3–virus interactions has expanded rapidly in both scope and conceptual complexity. Mechanistic studies have identified diverse, and often virus-specific, routes by which viral products activate or evade the NLRP3 inflammasome (Yue et al., 2024). More recently, host-associated modifiers, exemplified by microbiota-derived acetate, have also been shown to regulate NLRP3-dependent antiviral defense (Niu et al., 2023). This expansion has produced a fragmented evidence base spanning virology, innate immunity, microbiology and drug discovery, making it difficult to determine the field’s developmental trajectory, dominant contributors and emerging frontiers through qualitative reviews alone. Bibliometric studies have successfully characterized publication trends, collaborative networks and research hotspots in the closely related field of influenza co-infection (Zhang et al., 2025). Such quantitative mapping is particularly relevant because the broader NLRP3 inhibitor pipeline remains clinically immature, with selective inhibitors still lacking robust clinical validation (Vande Walle and Lamkanfi, 2024; Cabral et al., 2025). To our knowledge, however, no dedicated bibliometric study has systematically mapped the global landscape of NLRP3 research in viral infections. A virus-focused bibliometric analysis is therefore warranted to clarify how this interdisciplinary field has evolved and where future mechanistic, collaborative and translational efforts might be directed.

Bibliometric analysis provides established quantitative tools for mapping and visualizing publication landscapes, research hotspots, and citation trajectories in biomedical research (Ninkov et al., 2022). To address this gap, we performed a dual-database bibliometric analysis of all relevant literature retrieved from the WoSCC and Scopus up to April 28, 2026. We conducted multi-dimensional scientometric analyses including annual publication trends, cross-national/institutional collaboration networks, journal co-citation, citation burst detection and keyword co-occurrence clustering. By integrating these bibliometric outputs with published experimental and clinical evidence, we summarize dominant contemporary research frontiers and propose directions for future mechanistic experiments, clinical study design, and international collaboration.

## 2 Materials and methods

### 2.1 Data collection

**Figure 1:**
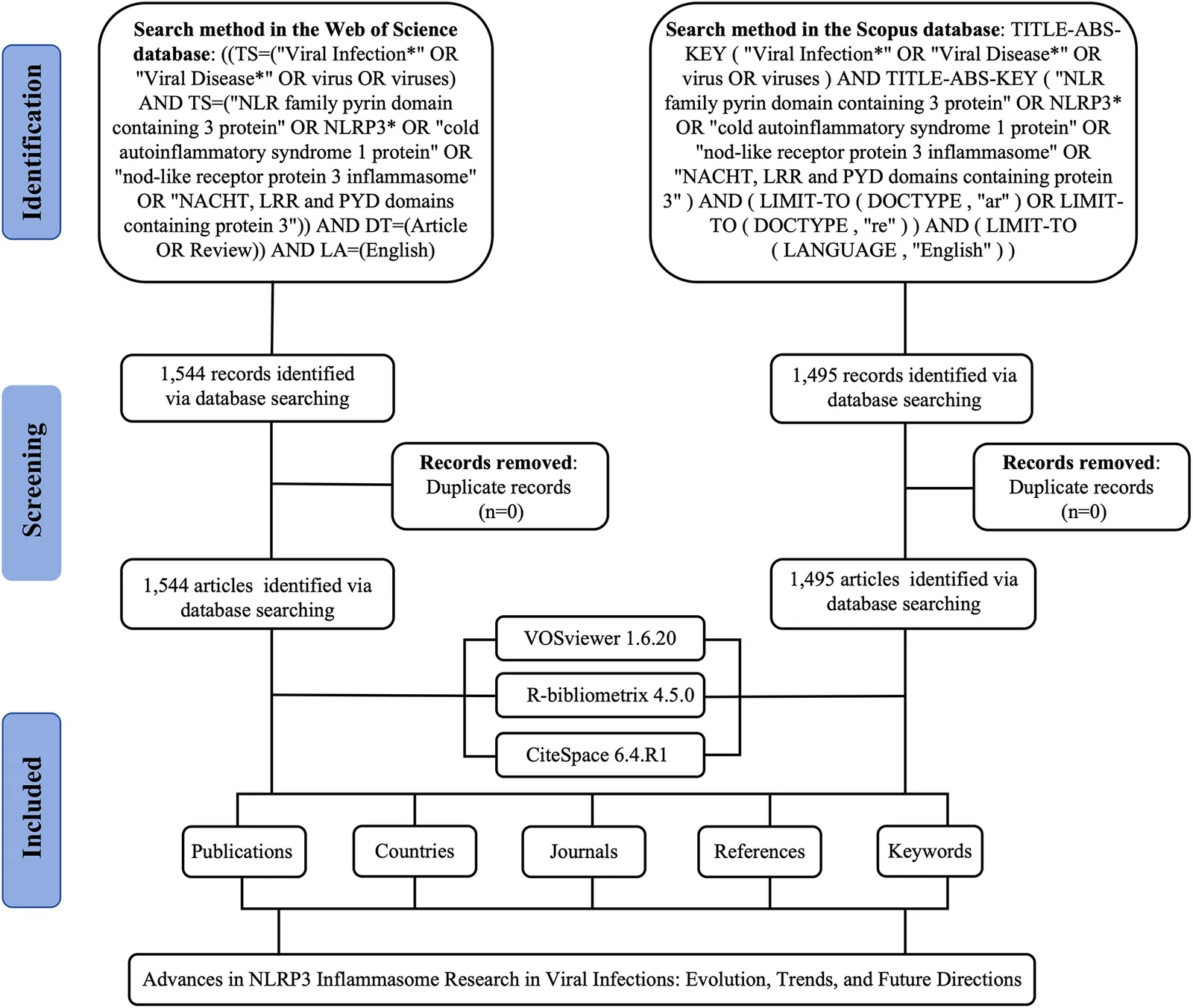
A flow chart of publication retrieval.

On April 28, 2026, we independently searched the Web of Science Core Collection (WoSCC) and Scopus. The specific search strategies are detailed in **Supplementary Annex 1** and **Figure 1**. Only English-language original articles and reviews were included. Records from WoSCC were exported as full records with cited references in plain-text format; records from Scopus were exported as full records with cited references in CSV format. Duplicates were removed in Excel by identifying records with identical digital object identifiers (DOIs). Each database was analyzed independently to preserve database-specific metadata and avoid information loss; cross-database comparisons were then performed narratively to identify convergent research hotspots.

### 2.2 Data analysis

Because WoSCC records provide more complete cited-reference metadata, which are required for co-citation and citation-burst analyses, our primary analyses were based on the WoSCC dataset. Results from the Scopus-based analysis, including annual publication trends and keyword clustering, are presented in **Supplementary Annexes 3** and **7** as cross-database validation.

Annual publication output was assessed using Origin 2018 software. Visual analyses and the generation of scientific knowledge maps were conducted using the bibliometrix package in R (R version 4.5.0) (Aria and Cuccurullo, 2017), VOSviewer (version 1.6.20) (van Eck and Waltman, 2010), and CiteSpace (version 6.4.R1) (Chen, 2006).

VOSviewer was used to visualize international collaboration networks, source co-citation patterns, and keyword co-occurrence. Journal-level metrics were visualized with ggplot2, and trend-topic analysis was performed with the bibliometrix package using author keywords. Journal impact factors (IFs) were sourced from the 2024 Journal Citation Reports (JCR). Detailed parameter settings for the VOSviewer analyses are outlined in **Supplementary Annex 2**. Because WoSCC and Scopus index keywords under different schemes, keyword counts were extracted separately for each database and integrated only narratively: themes were considered convergent when supported by both datasets, and no pooled keyword counts were computed.

## 3 Results

### 3.1 Annual scientific publications

**Figure 2:**
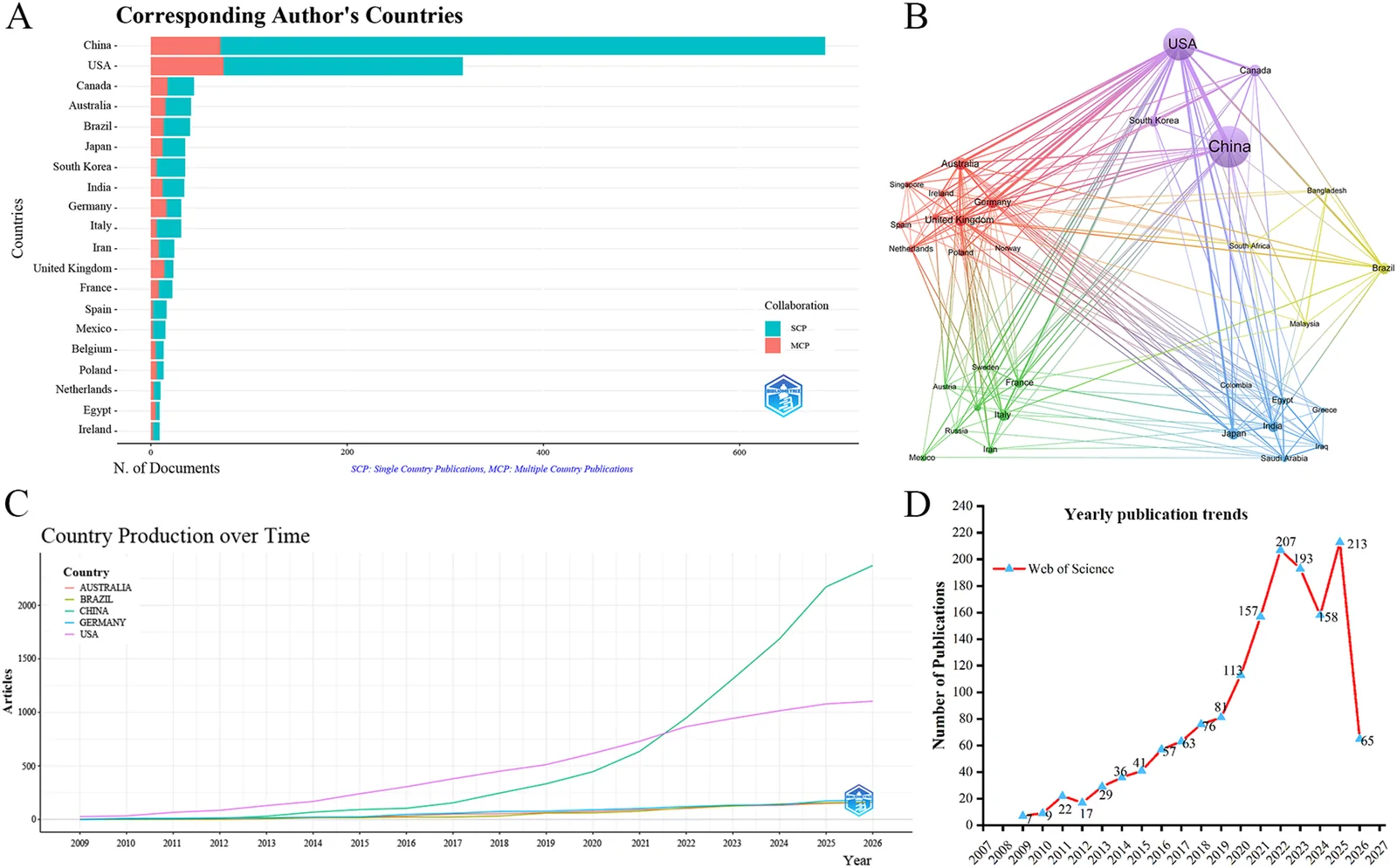
Annual publication trends and international collaboration in NLRP3 inflammasome research in viral infections. **(A)** Distribution of corresponding authors by country and collaborative patterns (SCP, single-country publication; MCP, multiple-country publication). **(B)** International collaboration map, with node size proportional to publication count. **(C)** Cumulative all-affiliation publication output of leading countries over time. **(D)** Yearly publication trends.

We identified 1,544 unique records in WoSCC. As shown in **Figure 2D**, annual output in this field increased overall from 2009 to 2025, with a particularly sharp rise between 2019 and 2022. Annual output remained below 30 articles throughout 2009–2013, moderated to 193 articles in 2023 and 158 in 2024, and rebounded to a peak of 213 articles in 2025. By April 28, 2026, 65 articles had already been published in 2026; as a partial-year count, this figure should not be interpreted as a decline in output.

A total of 1,495 unique records were retrieved from Scopus. The temporal growth pattern closely resembled that seen in WoSCC, exhibiting a rapid increase that commenced around 2019, culminating in a peak of 220 articles in 2025; the lower count recorded for 2026 reflects the incomplete publication year at the search date rather than a decline in output (**Supplementary Annex 3A**). This concordant upward trend across both databases suggests sustained interest in NLRP3 inflammasome research in the context of viral infections over the past decade; the small numerical difference between the two databases is expected given differences in database coverage.

### 3.2 Major countries/regions and institutions

Research on the NLRP3 inflammasome in viral infections is concentrated in a few leading countries (**Table 1**; **Figure 2A**). China ranks first with 687 publications (44.5%), followed by the United States with 318 (20.6%); smaller but steady contributions came from Canada, Australia, Brazil, Japan, South Korea, India, and Germany (**Table 1**). High-income countries such as Germany and the United Kingdom have a notable proportion of internationally co-authored articles, whereas China shows a lower multiple-country publication (MCP) percentage, indicating predominantly domestic authorship. In the international collaboration network (**Figure 2B**), the United States occupies a central hub position connecting Europe, Canada, and Australia, whereas China forms the largest node with predominantly domestic co-authorship links.

**Table 1:** Most relevant countries/regions of corresponding authors in NLRP3 inflammasome research on viral infections.

Country	Articles	Articles (%)	SCP	MCP	MCP (%)
China	687	44.5	616	71	10.3
USA	318	20.6	244	74	23.3
Canada	44	2.8	27	17	38.6
Australia	41	2.7	26	15	36.6
Brazil	40	2.6	27	13	32.5
Japan	35	2.3	23	12	34.3
South Korea	35	2.3	29	6	17.1
India	34	2.2	22	12	35.3
Germany	31	2	15	16	51.6
Italy	31	2	25	6	19.4
Iran	24	1.6	16	8	33.3
United Kingdom	23	1.5	9	14	60.9
France	22	1.4	14	8	36.4
Spain	16	1	14	2	12.5
Mexico	15	1	13	2	13.3
Belgium	13	0.8	8	5	38.5
Poland	13	0.8	7	6	46.2
Netherlands	10	0.6	7	3	30
Egypt	9	0.6	4	5	55.6
Ireland	9	0.6	7	2	22.2
Switzerland	9	0.6	3	6	66.7
Colombia	7	0.5	5	2	28.6
Greece	7	0.5	5	2	28.6
Malaysia	5	0.3	2	3	60
Singapore	5	0.3	2	3	60

MCP, Multiple country publication; SCP, Single country publication.

Temporal publication trends of leading nations (**Figure 2C**) indicate modest output before 2013, followed by rapid growth after 2015. China and the USA show the most pronounced increases, including a marked surge in Chinese output after 2019. Importantly, **Figure 2C** plots cumulative counts based on all author affiliations, which differ in scope from the corresponding-author counts shown in **Figure 2A** and **Table 1**; on this cumulative all-affiliation basis, China’s output surpassed 2,000 articles by 2026. Australia, Brazil, and Germany also show consistent growth, albeit at lower levels. These curves suggest a swift global research expansion led primarily by China and the USA (**Figure 2C**).

**Figure 3:**
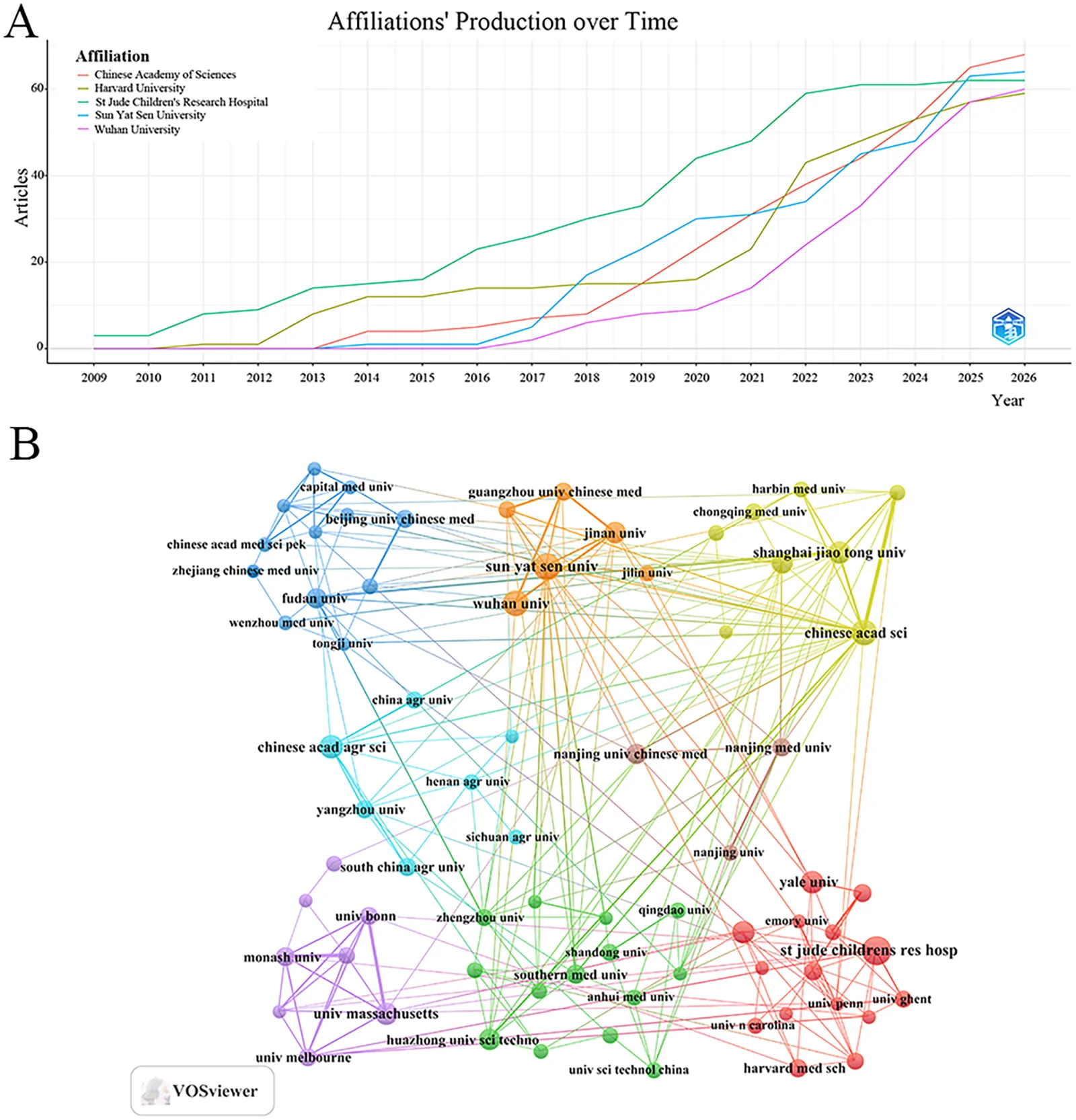
Institutional distribution and collaborative networks in NLRP3 inflammasome research. **(A)** Cumulative publication output of leading institutions over time. **(B)** Institutional collaboration map, with node size proportional to publication count.

The most productive institutional affiliations (**Figure 3**; **Table 2**) are the Chinese Academy of Sciences (n = 68), Sun Yat-sen University (n = 64), St. Jude Children’s Research Hospital (n = 62), Wuhan University (n = 60), and Harvard University (n = 59). Output from these centers has steadily increased, particularly after 2018. Among leading affiliations (**Figure 3A**), St. Jude Children’s Research Hospital established the earliest sustained lead, Chinese institutions accelerated after approximately 2018–2020, Wuhan University displays the steepest post-2020 cumulative curve, and the Chinese Academy of Sciences reached first place by 2025–2026. Chinese academic institutions (such as the Chinese Academy of Sciences, Sun Yat-sen University, and Wuhan University) and leading North American institutions (including St. Jude Children’s Research Hospital and Harvard University) form closely linked clusters in the collaboration network, suggesting the emergence of multiple high-output hubs that shape the field’s output (**Figure 3B**).

**Table 2:** Most relevant author affiliations in NLRP3 inflammasome research on viral infections.

Affiliation	Articles
Chinese Academy of Sciences	68
Sun Yat-sen University	64
St. Jude Children’s Research Hospital	62
Wuhan University	60
Harvard University	59
Yale University	54
University of California System	51
Fudan University	47
Nanjing University of Chinese Medicine	44
Jinan University	43

### 3.3 Journals and co-cited journals

**Figure 4:**
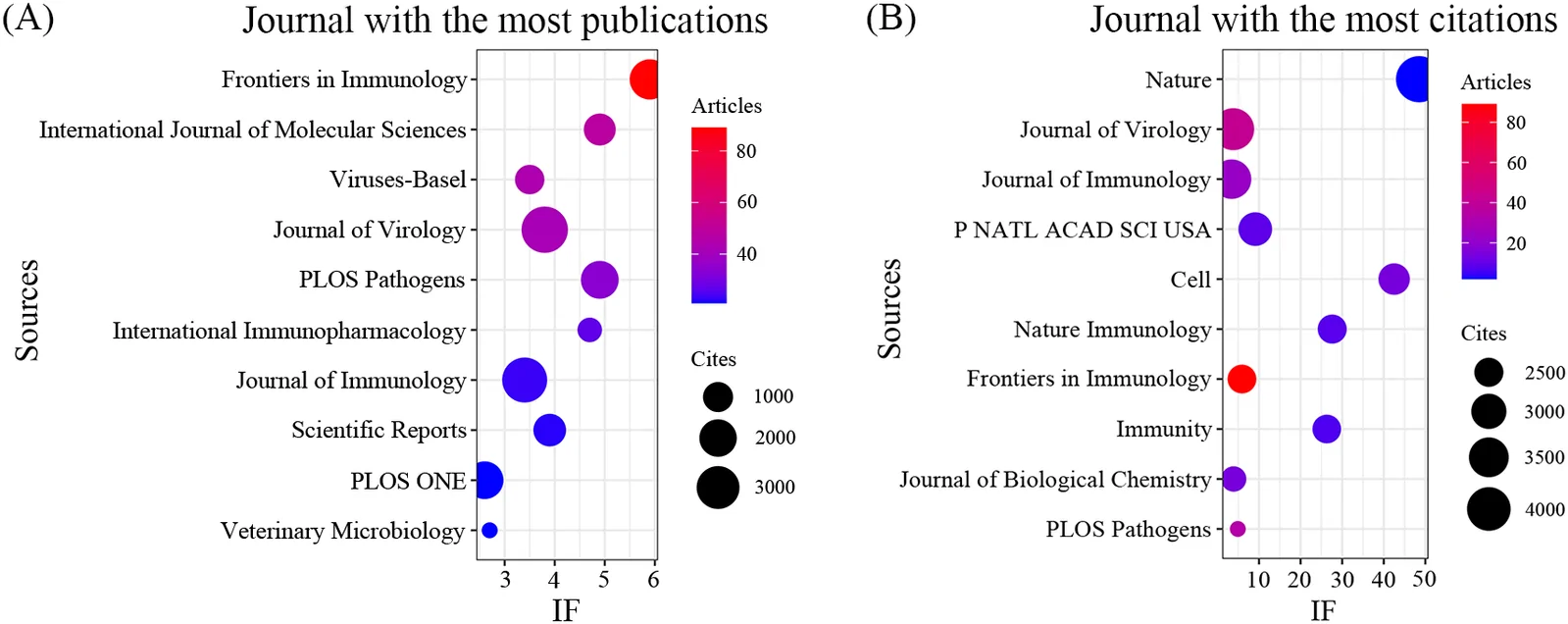
Journal productivity and citation influence in NLRP3 inflammasome research on viral infections. **(A)** Journal with the most publications. **(B)** Journal with the most citations.

**Figure 5:**
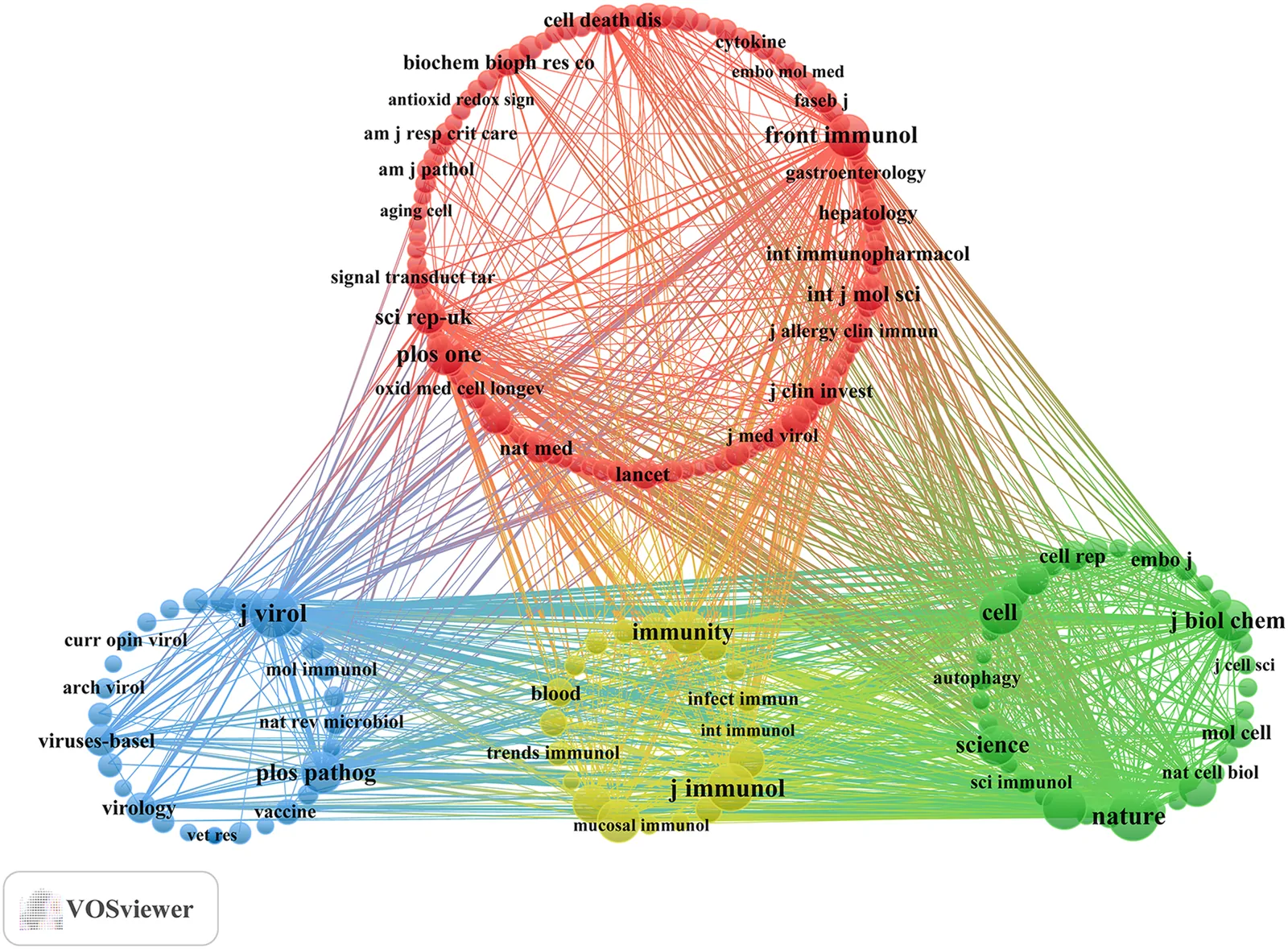
Co-citation analysis of journals on NLRP3 inflammasome research in viral infections.

The 1,544 WoSCC articles were published across 521 journals (**Supplementary Annex 4**). The five most productive journals were Frontiers in Immunology (89 articles, IF = 5.9), International Journal of Molecular Sciences (48, IF = 4.9), Viruses-Basel (44, IF = 3.5), Journal of Virology (42, IF = 3.8), and PLOS Pathogens (34, IF = 4.9), as shown in **Figure 4A**; **Table 3**. **Figure 4B** and **Table 4** present the five most cited journals. Nature received the most citations (4,374, IF = 48.5), followed by Journal of Virology (3,763, IF = 3.8), Journal of Immunology (3,457, IF = 3.4), Proceedings of the National Academy of Sciences of the United States of America (2,855, IF = 9.1), and Cell (2,672, IF = 42.5). The co-citation map suggests that Frontiers in Immunology, Nature, Journal of Virology, and Journal of Immunology serve as the primary hubs in the journal network, consistent with a central role in connecting research on NLRP3 inflammasome and viral infections (**Figure 5**).

**Table 3:** Top 10 most productive journals.

Journal	Documents	Cites	IF (2024)
Frontiers in Immunology	89	2477	5.9
International Journal of Molecular Sciences	48	1215	4.9
Viruses-Basel	44	932	3.5
Journal of Virology	42	3763	3.8
PLOS Pathogens	34	2060	4.9
International Immunopharmacology	27	517	4.7
Journal of Immunology	23	3457	3.4
Scientific Reports	22	1301	3.9
PLOS ONE	21	2039	2.6
Veterinary Microbiology	21	236	2.7

**Table 4:** Top 10 most cited journals.

Journal	Cites	Documents	IF (2024)
Nature	4374	2	48.5
Journal of Virology	3763	42	3.8
Journal of Immunology	3457	23	3.4
P NATL ACAD SCI USA	2855	9	9.1
Cell	2672	14	42.5
Nature Immunology	2497	8	27.6
Frontiers in Immunology	2477	89	5.9
Immunity	2471	7	26.3
Journal of Biological Chemistry	2286	14	3.9
PLOS Pathogens	2060	34	4.9

P NATL ACAD SCI USA, Proceedings of the National Academy of Sciences of the United States of America.

### 3.4 Analysis of cited papers on the NLRP3 inflammasome in viral infections

#### 3.4.1 Top 25 most cited articles

We employed the bibliometrix package in R to identify the top 25 cited articles on NLRP3 inflammasomes in viral infections (**Table 5**). Each of these papers has received at least 460 citations, and they collectively represent much of the field’s foundational knowledge base. These 25 articles are spread among 18 different journals, suggesting a diverse journal landscape without a dominant primary publication venue. The journal Immunity leads with 4 articles (Allen et al., 2009; Thomas et al., 2009; Guarda et al., 2011; Kawai and Akira, 2011), followed by Nature Immunology and Immunological Reviews, each with 3 articles. Other journals such as Nature Reviews Cardiology, Nature Communications, and Proceedings of the National Academy of Sciences of the United States of America are each represented by a single article. Overall, the highly cited literature predominantly appears in leading immunology and high-impact general science journals. The top three cited references are “Toll-like Receptors and Their Crosstalk with Other Innate Receptors in Infection and Immunity” (Kawai and Akira, 2011); “Inflammageing: chronic inflammation in ageing, cardiovascular disease, and frailty” (Ferrucci and Fabbri, 2018); and “Molecular mechanisms and functions of pyroptosis, inflammatory caspases and inflammasomes in infectious diseases” (Man et al., 2017).

**Table 5:** Top 25 most cited references on NLRP3 inflammasome in viral infections.

Paper	DOI	Total citations	TC per year
KAWAI T, 2011, IMMUNITY	10.1016/j.immuni.2011.05.006	2996	187.25
FERRUCCI L, 2018, NAT REV CARDIOL	10.1038/s41569-018-0064-2	2718	302.00
MAN SM, 2017, IMMUNOL REV	10.1111/imr.12534	1383	138.30
ROGERS C, 2017, NAT COMMUN	10.1038/ncomms14128	1248	124.80
ICHINOHE T, 2011, P NATL ACAD SCI USA	10.1073/pnas.1019378108	1194	74.63
JO EK, 2016, CELL MOL IMMUNOL	10.1038/cmi.2015.95	1162	105.64
ROEHLEN N, 2020, CELLS-BASEL	10.3390/cells9040875	985	140.71
MIAO EA, 2011, IMMUNOL REV	10.1111/j.1600-065X.2011.01044.x	960	60.00
ALLEN IC, 2009, IMMUNITY	10.1016/j.immuni.2009.02.005	955	53.06
HEYMANN F, 2016, NAT REV GASTRO HEPAT	10.1038/nrgastro.2015.200	933	84.82
JORGENSEN I, 2017, NAT REV IMMUNOL	10.1038/nri.2016.147	812	81.20
GUARDA G, 2011, IMMUNITY	10.1016/j.immuni.2011.02.006	780	48.75
MUELLER AL, 2020, AGING-US	10.18632/aging.103344	746	106.57
KURIAKOSE T, 2016, SCI IMMUNOL	10.1126/sciimmunol.aag2045	699	63.55
CHANG YJ, 2011, NAT IMMUNOL	10.1038/ni.2045	662	41.38
THOMAS PG, 2009, IMMUNITY	10.1016/j.immuni.2009.02.006	637	35.39
GALLUZZI L, 2017, ANNU REV PATHOL-MECH	10.1146/annurev-pathol-052016-100247	590	59.00
ICHINOHE T, 2009, J EXP MED	10.1084/jem.20081667	579	32.17
NIIKURA K, 2013, ACS NANO	10.1021/nn3057005	549	39.21
ICHINOHE T, 2010, NAT IMMUNOL	10.1038/ni.1861	545	32.06
YAO CX, 2018, CIRCULATION	10.1161/CIRCULATIONAHA.118.035202	484	53.78
ZHENG M, 2020, IMMUNOL REV	10.1111/imr.12909	476	68.00
GROOTJANS S, 2017, CELL DEATH DIFFER	10.1038/cdd.2017.65	474	47.40
DIAMOND MS, 2022, NAT IMMUNOL	10.1038/s41590-021-01091-0	471	94.20
CHRISTGEN S, 2020, FRONT CELL INFECT MI	10.3389/fcimb.2020.00237	460	65.71

TC, total citations.

#### 3.4.2 Top 25 references with the strongest citation bursts

**Figure 6:**
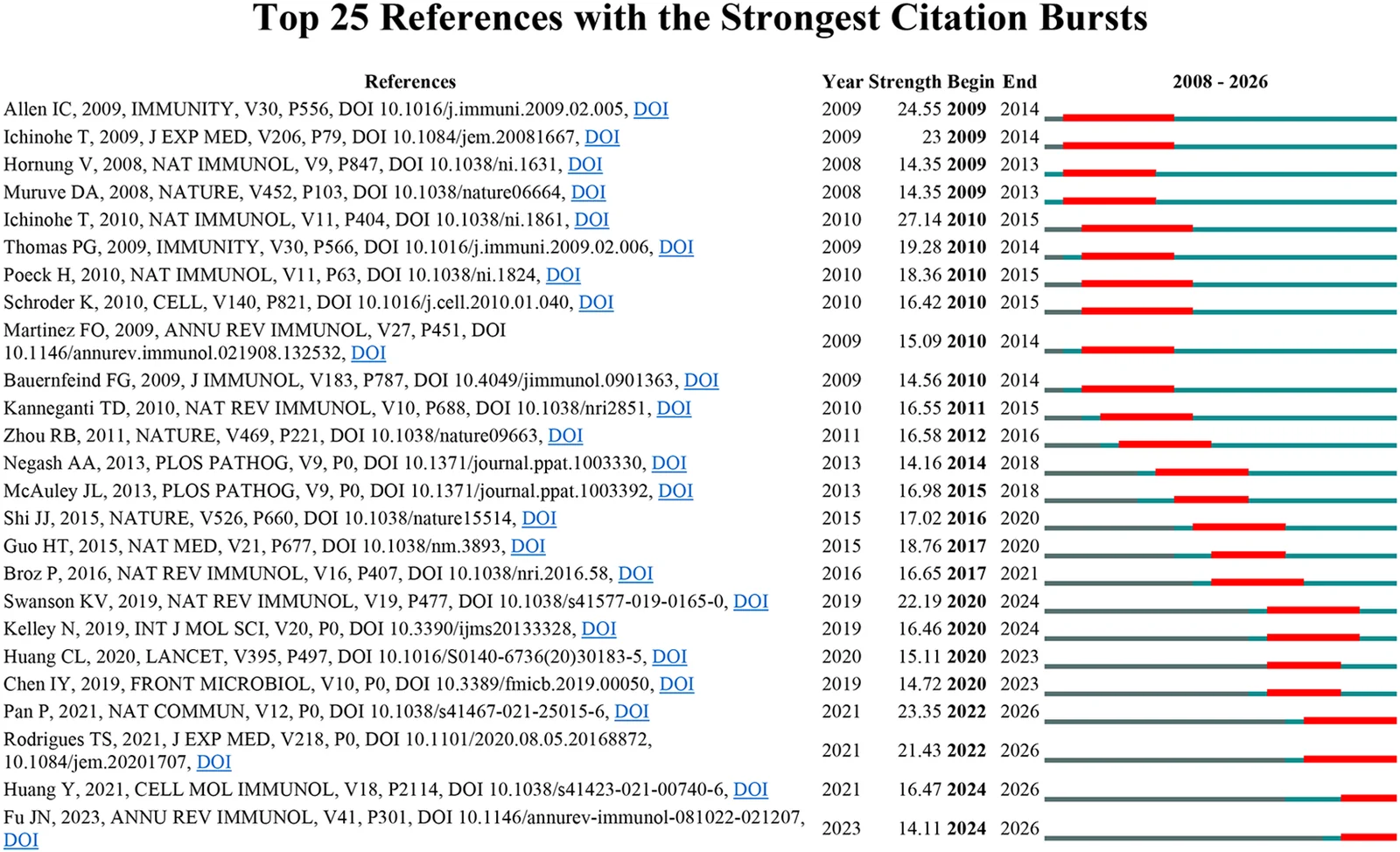
Top 25 references with the strongest citation bursts on NLRP3 inflammasome in viral infections. The “References” column on the left lists the first author, publication year, and source journal of each reference; the “Strength” column reports the numeric citation burst strength; and “Begin” and “End” indicate the onset and termination years of each burst, respectively. On the timeline, green segments represent the baseline citation level, whereas red segments indicate the active burst period during which citations to the reference surged, with the length of each red segment directly reflecting the burst duration (in years). This visualization enables rapid identification of the core highly influential references within different periods and illustrates the life cycle of their academic impact. DOI identifiers are included for direct access to the source materials, and the complete list is provided in **Supplementary Annex 5**.

Using CiteSpace, we identified 354 references with significant citation bursts; the 25 strongest are shown in **Figure 6**, and the complete list with DOIs is provided in **Supplementary Annex 5**. Because burst strength quantifies the acceleration of citations over a defined period, these values are interpreted here as markers of research attention rather than of the methodological quality of the cited references. The top three references with the strongest citation bursts were as follows: “Influenza virus activates inflammasomes via its intracellular M2 ion channel” (strength: 27.14), “The NLRP3 Inflammasome Mediates *In Vivo* Innate Immunity to Influenza A Virus through Recognition of Viral RNA” (strength: 24.55), and “SARS-CoV-2 N protein promotes NLRP3 inflammasome activation to induce hyperinflammation” (strength: 23.35). These studies collectively highlight the role of influenza virus components (M2 ion channel and viral RNA) and SARS-CoV-2 N protein in triggering NLRP3 inflammasome activation, with implications for antiviral responses and inflammation and for potential NLRP3-targeted interventions.

Among the more recent bursts, Rodrigues et al., “Inflammasomes are activated in response to SARS-CoV-2 infection and are associated with COVID-19 severity in patients” (strength: 21.43), Huang et al., “NLRP3 inflammasome activation and cell death” (strength: 16.47), and Fu and Wu, “Structural mechanisms of NLRP3 inflammasome assembly and activation” (strength: 14.11) further extended this trajectory. These recent bursts indicate a close association between SARS-CoV-2-induced inflammasome activation, COVID-19 severity, and hyperinflammation, point to the involvement of NLRP3 in inflammatory tissue damage via regulated cell-death pathways, and provide a structural basis for ongoing efforts to develop NLRP3-targeted therapies.

Based on the 25 references with the strongest citation bursts, current research on the NLRP3 inflammasome in viral infections can be grouped into three main areas: (1) elucidating the various molecular mechanisms through which viruses activate NLRP3, including ion channels, viral proteins, nucleic acids, and organelle damage; (2) exploring the dual function of NLRP3 in antiviral immunity, acting both as a mediator of host defense and a driver of excessive inflammation and pathological damage; (3) investigating the molecular interplay between NLRP3 inflammasomes and cell death, along with their potential as therapeutic targets. These research areas point to shared immunopathological pathways from influenza virus to SARS-CoV-2 and may inform future work on antiviral treatments and vaccine adjuvant development.

### 3.5 Analysis of keywords in NLRP3 inflammasome research in viral infections

#### 3.5.1 Co-occurrence analysis of keywords

**Figure 7:**
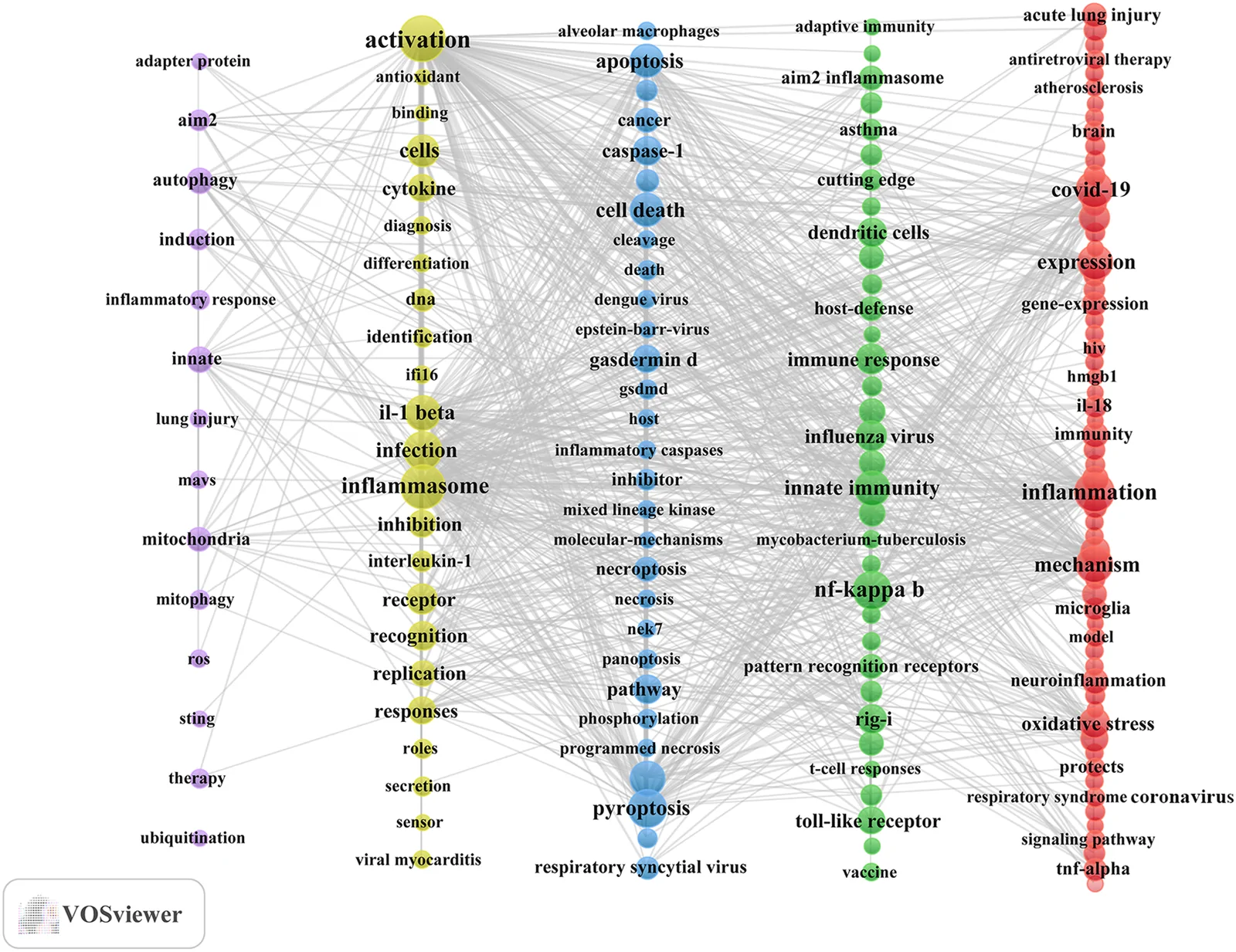
Keyword co-occurrence map of NLRP3 inflammasome in viral infections publications.

Keyword co-occurrence analysis is widely used to identify research trends in a field. Using VOSviewer, we detected 6,039 keywords in the analyzed publications, of which 20 appeared more than 86 times; the most frequent were “activation” (n = 375), “inflammasome” (n = 316), “inflammation” (n = 223), “NF-κB” (nuclear factor kappa B; n = 208), and “pyroptosis” (n = 200) (**Table 6**). With a minimum occurrence threshold of 12, 197 keywords were retained and grouped into five clusters, each shown in a distinct color (**Figure 7**). Because not all cluster terms appear as labels in **Figure 7**, the representative terms listed below are drawn from the full keyword lists underlying each cluster (**Supplementary Annex 6**). Nevertheless, it must be noted that the frequency of keyword co-occurrence reflects only the publication attention toward a topic and cannot be directly equated with experimental power or the grade of clinical evidence.

**Table 6:** Top 20 keywords related to NLRP3 inflammasome in viral infections.

Rank	Keywords	Count
1	activation	375
2	inflammasome	316
3	inflammation	223
4	nf-kappa b	208
5	pyroptosis	200
6	infection	189
7	covid-19	165
8	mechanism	164
9	innate immunity	158
10	il-1 beta	154
11	protein	151
12	expression	147
13	apoptosis	129
14	cell death	125
15	cells	118
16	disease	98
17	receptor	97
18	oxidative stress	93
19	influenza virus	88
20	immune response	87

Cluster 1 (red) centers on NLRP3 inflammasome-mediated multiorgan inflammation and viral disease phenotypes, with particular emphasis on COVID-19-related acute lung injury, cytokine storm, neuroinflammation, liver pathology, and biomarker-based risk stratification. Representative terms in this cluster include COVID-19, coronavirus, acute lung injury, cytokine storm, neuroinflammation, fibrosis, biomarker, and susceptibility.

Cluster 2 (green) focuses on innate and adaptive antiviral immunity and delineates how pattern-recognition receptors, type I interferon signaling, and vaccine or adjuvant responses shape host defense against viral infections, especially influenza and other respiratory viruses. Key terms in this cluster comprise innate immunity, immune response, interferon, pattern recognition receptors, retinoic acid-inducible gene I (RIG-I), Toll-like receptors, influenza virus, vaccine, and adjuvant.

Cluster 3 (blue) maps inflammasome-associated regulated cell death pathways during viral infection, highlighting the NLRP3-caspase-1-GSDMD axis and its interplay with apoptosis, necroptosis, and pyroptosis-apoptosis-necroptosis (PANoptosis) across a broad range of viral pathogens. Representative terms include pyroptosis, apoptosis, cell death, caspase-1, gasdermin D, necroptosis, PANoptosis, respiratory syncytial virus, and dengue virus.

Cluster 4 (yellow) highlights core inflammasome signaling, describing canonical NLRP3 activation, IL-1β-driven inflammatory cascades, host-pathogen recognition, and the interaction between inflammasome activity and viral replication, with growing implications for diagnosis and targeted therapeutic inhibition. Dominant terms include activation, inflammasome, IL-1β, infection, receptor, recognition, replication, and viral myocarditis.

Cluster 5 (purple) relates to mitochondria-associated innate sensing and cellular quality-control mechanisms and illustrates how autophagy/mitophagy, mitochondrial antiviral-signaling protein (MAVS) and stimulator of interferon genes (STING) signaling, reactive oxygen species generation, and ubiquitination may collectively regulate NLRP3 inflammasome activation in virus-induced tissue injury, consistent with experimental evidence linking mitochondrial dysfunction, autophagy, and MAVS/STING pathways to NLRP3 regulation (Nakahira et al., 2011; Zhou et al., 2011; Subramanian et al., 2013; Gaidt et al., 2017). Key terms in this cluster include mitochondria, autophagy, mitophagy, MAVS, STING, ROS, ubiquitination, lung injury, and therapy.

The full list of keywords in these clusters is available in **Supplementary Annex 6**. Additionally, using the Scopus dataset, we extracted a total of 14,459 keywords. Applying a minimum occurrence threshold of 52, we identified 197 keywords and generated a keyword cluster map (**Supplementary Annex 3B**), which depicts four distinct clusters, each represented by a different color.

#### 3.5.2 Cross-database keyword co-occurrence analysis from Web of Science and Scopus

Keyword co-occurrence maps from Web of Science (WoS; **Figure 7**; **Supplementary Annex 6**) and Scopus (**Supplementary Annexes 3B**, **7**) identified prominent research areas at the intersection of NLRP3 inflammasomes and viral infections. Because keyword vocabularies differ between databases, the maps were compared narratively rather than by pooled counts. Unless otherwise specified, unlabeled keyword frequencies in this subsection refer to Scopus; Web of Science–derived counts are labeled explicitly. Recurrent themes included disease impact, experimental approaches, molecular pathways, clinical applications, and therapeutic strategies.

1. Priority disease models and experimental systems: Research in this field relies heavily on *in vitro* and *in vivo* models. *In vitro* studies mainly use human cell lines and primary cells, including the “THP-1 cell line” and “peripheral blood mononuclear cell”; “human cell” (n = 380) is closely linked to clinical settings through “human” (n = 1,016) and “clinical article” (n = 76). *In vivo* research focuses on mouse models, as reflected in terms such as “mice” (n = 611), “animal experiment” (n = 616), “animal model” (n = 551), and specific strains such as “C57BL mouse” (n = 229) and “Bagg albino mouse” (n = 52). These models are commonly used alongside genetic manipulations such as “knockout mouse” (n = 105), “gene overexpression” (n = 102), and “small interfering RNA” (n = 98), emphasizing a mechanistic approach in experimental design. At the population level, age and comorbidities are not strictly defined; human data are instead characterized by “adult” (n = 210) and “middle aged” (n = 67), with outcome measures concentrating on “disease severity” (n = 88) and “mortality” (n = 53).
2. Viral triggers and co-infection patterns: The dominant virus-related terms are “influenza” and “influenza A virus”, followed by “COVID-19” (Scopus: n = 218; WoS: n = 165), “human immunodeficiency virus type 1 (HIV-1)”/”HIV infections” (n = 82), “respiratory syncytial virus” (n = 32), “dengue virus” (n = 15), “hepatitis C virus” (n = 15), and “Epstein-Barr virus” (n = 13). Experimental studies have linked four of these pathogens—respiratory syncytial virus, dengue virus, hepatitis C virus, and HIV-1—to NLRP3 inflammasome activation (Segovia et al., 2012; Negash et al., 2013; Wu et al., 2013; Rawat et al., 2019). The terms “coronavirus” (n = 26) and “respiratory syndrome coronavirus” (n = 21) suggest a connection between pre-COVID coronavirus research and current SARS-related literature. Co-infection is mostly implicit: viral and bacterial terms co-occur (e.g., “mycobacterium-tuberculosis” in the WoS clusters) but are rarely reported as explicit dual-pathogen combinations, in contrast to influenza-focused research. Notably, “viral myocarditis” (n = 14) and “hepatocellular-carcinoma” (n = 17) point to a possible association between NLRP3 activation, organ-specific damage, and virus-induced tumorigenesis.
3. Molecular mechanisms: inflammasome activation, pyroptosis, and cell-death pathways: This domain is extensively researched, with key nodes being “inflammasome activation” (n = 56), “caspase-1 activation” (n = 26), and “pyroptosis” (WoS: n = 200; Scopus: n = 352). Mechanistic terms such as “gasdermin D” (n = 66), “GSDMD” (n = 21), “necroptosis” (n = 42), “PANoptosis” (n = 20), “apoptosis” (Scopus: n = 275), and “cell death” (WoS: n = 125) collectively outline the NLRP3-caspase-1-GSDMD axis and its interactions with various regulated cell-death processes. Upstream signals include “NF-κB” (WoS: n = 208; Scopus: n = 144), “RIG-I” (n = 73), “Toll-like receptor” (n = 59), and “absent in melanoma 2 (AIM2) inflammasome” (n = 40), highlighting the substantial roles of pattern-recognition receptors and transcription factors in inflammasome activation. Downstream effectors are well represented by IL-1 (Scopus: n = 823) and IL-1β (WoS: n = 154; Scopus: n = 80); IL-18 (WoS: n = 34; Scopus: n = 393) and tumor necrosis factor-α (TNF-α; n = 38) are also represented. Regulatory elements include “autophagy” (n = 55), “mitochondria” (n = 65), “reactive oxygen species” (WoS: n = 14; Scopus: n = 230), “ubiquitination” (n = 12), and “STING” (n = 12), collectively underscoring the potential significance of mitochondrial homeostasis, autophagy, and oxidative stress in modulating NLRP3 signaling. These findings collectively point to the NLRP3-caspase-1-GSDMD pyroptotic axis as a central mechanistic hub.
4. Inflammation-related clinical and translational biomarkers: Common terms related to human disease are “inflammation” (Scopus: n = 579; WoS: n = 223), “cytokine production” (n = 196), “cytokine release” (n = 205), “cytokine storm” (Scopus: n = 71; WoS: n = 42), and “oxidative stress” (Scopus: n = 154; WoS: n = 93). Possible biomarkers include “biological marker” (n = 56), “IL-18” (Scopus: n = 393; WoS: n = 34), “HMGB1” (high mobility group box 1; n = 13), and “neutrophil extracellular traps” (n = 21). Diseases range from acute organ damage to chronic inflammation and degenerative conditions, such as “acute lung injury” (n = 39), “pneumonia” (Scopus: n = 60; WoS: n = 18), “fibrosis” (n = 28), “atherosclerosis” (n = 15), “Alzheimer’s disease” (n = 34), and “neuroinflammation” (Scopus: n = 116; WoS: n = 49). Moreover, “cancer” (n = 41) and “hepatocellular-carcinoma” (n = 17) suggest a potential link of NLRP3 to virus-induced cancer development. Overall, IL-1β, IL-18, cytokine storm, and oxidative stress markers are increasingly studied as candidate indicators for evaluating disease severity and prognosis in viral infections.
5. Therapeutic targeting and intervention strategies: Therapy-related terms are increasingly prominent, with keywords such as “inhibitor” (n = 25), “antiviral agent” (Scopus: n = 95), “anti-inflammatory activity” (n = 109), “drug efficacy” (n = 54), and “drug mechanism” (n = 70). “Therapy”-related terms were also notable (WoS: n = 19; in Scopus, the co-occurrence of “autophagy” and “therapy” reached n = 55). Specific interventions include “vaccine” (n = 17), “antiretroviral therapy” (n = 19), and “nanoparticles” (n = 17), while “gene knockdown” (n = 66) and “small interfering RNA” (n = 98) represent gene-based approaches. Advanced NLRP3 inhibitors such as MCC950 are not yet common terms, suggesting a focus on preclinical stages. However, “signal transduction” (n = 535) and “drug mechanism” (n = 70) highlight continued interest in refining NLRP3 signaling for future therapies.

#### 3.5.3 Trend topics analysis

**Figure 8:**
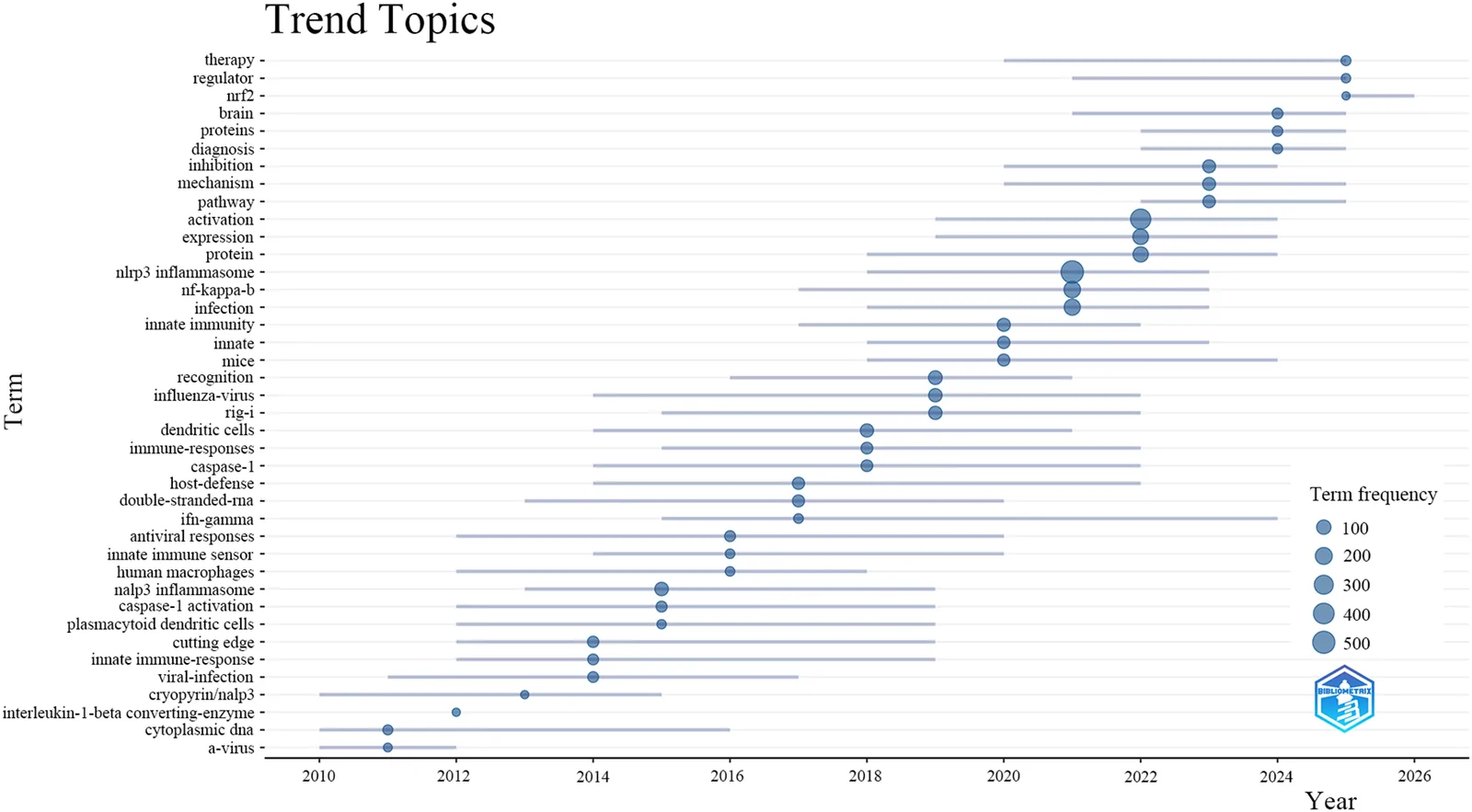
Trending topics in NLRP3 inflammasome research on viral infections.

A trend-topic map generated with the bibliometrix package in R traces the chronological evolution of research focus in this field (**Figure 8**). Between 2010 and 2014, trending terms centered on the fundamental components of inflammasome activation, such as cryopyrin/NALP3, caspase-1 activation, interleukin-1β converting enzyme, and innate immune sensing of cytoplasmic DNA. From 2014 to 2018, the emphasis shifted toward influenza A virus recognition, RIG-I, host-defense mechanisms, dendritic-cell responses, double-stranded RNA sensing, interferon-γ (IFN-γ), and antiviral responses in human macrophages. Between 2018 and 2022, the dominant topics were the NLRP3 inflammasome itself, the NF-κB pathway, innate immunity in mouse models, and the interplay between viral infection and inflammasome-driven immune responses. From 2022 to 2026, emerging terms—including therapeutic targeting of the NLRP3 inflammasome, pathway- and regulator-related terms, regulatory molecules such as nuclear factor erythroid 2-related factor 2 (NRF2), mechanisms of protein expression and inhibition, diagnosis, and brain-related pathology (the trend term “brain”)—indicate a translational turn. Overall, this chronological progression suggests an evolution from basic molecular mechanisms toward disease-oriented pathological research and, more recently, toward potential therapeutic applications.

## 4 Discussion

### 4.1 General information

This study provides a dual-database bibliometric mapping of NLRP3 inflammasome research in viral infections, integrating 1,544 unique records from the WoSCC with 1,495 unique records from Scopus retrieved through April 28, 2026. Previous NLRP3 bibliometric analyses have either surveyed the broader, most highly cited NLRP3 field without distinguishing viral from non-viral contexts (Chen et al., 2026), or relied on single databases and specific disease domains such as heart failure (Lian et al., 2025) or acute lung injury/acute respiratory distress syndrome (Xiao et al., 2022). By combining cross-dataset validation with systematic mapping, our analysis delineates how this field has been structured and reshaped over nearly two decades. In this section, we interpret the temporal, geographical, institutional, and journal-level patterns that define the field’s structural features (Results 3.1–3.3).

The annual publication curve (**Figure 2D**) shows an overall increase from 2009 to 2025, within which several phases can be discerned. Output remained low from 2009 to 2013, corresponding to the field’s foundational period, when mechanistic studies of influenza-driven NLRP3 activation established its conceptual basis (Allen et al., 2009; Thomas et al., 2009). Between 2014 and 2019, output rose steadily, reflecting a broadening of interest from influenza toward a wider spectrum of viral infections. The steepest expansion occurred in 2019–2022, coinciding with the COVID-19 pandemic—likely the field’s most influential public-health event during the study period. This association is biologically plausible: the SARS-CoV-2 nucleocapsid protein can promote NLRP3 inflammasome assembly and hyperactivation, linking it to the hyperinflammation of severe COVID-19 (Pan et al., 2021), and a comparable surge occurred in NLRP3 research on acute lung injury (Xiao et al., 2022). Although output moderated in 2023–2024 before rebounding to a new peak in 2025 in both databases (Results 3.1), it remained well above pre-pandemic levels, suggesting that the pandemic durably expanded the field rather than producing a transient spike. The lower 2026 count reflects the incomplete publication year at the search date and should not be interpreted as declining interest.

At the country level, the corresponding-author distribution (**Figure 2A**; **Table 1**) is dominated by China and the United States, which together account for roughly two-thirds of publications; their collaboration profiles, however, differ markedly, with China showing a much lower multi-country publication (MCP) ratio (**Table 1**). Because **Figure 2A** and **Table 1** count corresponding authors, whereas **Figure 2C** aggregates cumulative output across all author affiliations, the two views are complementary rather than contradictory: the United States led cumulative production before the pandemic, China overtook it around 2021–2022, and by 2026 its cumulative curve had reached more than double that of the United States. This crossover is consistent with the long-term expansion of Chinese research capacity, investment, and institutional growth documented at the national level (Xie et al., 2014; Tollefson, 2018), and with recent shifts in China’s research assessment system (Zhang and Sivertsen, 2020), which may have channeled additional effort toward rapidly growing fields. The timing of the acceleration further suggests a pandemic-related component: the early outbreak centered in Wuhan (Huang et al., 2020) created urgent domestic demand for respiratory-virus research in China, and bibliometric studies of the early pandemic period show that coronavirus research expanded rapidly, largely by consolidating established collaboration networks (Fry et al., 2020; Cai et al., 2021) and by drawing an unprecedented influx of researchers into virus-related topics (Ioannidis et al., 2021). The collaboration network (**Figure 2B**) adds a structural qualification to this volume-based leadership: the United States functions as a broad hub connecting Europe, Canada, and Australia, whereas China forms the largest node but with dense, predominantly domestic linkages—a pattern also reported in NLRP3 cardiovascular research (Lian et al., 2025) and consistent with the asymmetric distribution of visibility and collaboration benefits documented for US–China scientific partnerships more generally (Lee and Haupt, 2020). Several mid-volume European countries display the highest MCP ratios (**Table 1**), suggesting that they occupy integrative positions in the network; because some of these ratios are based on small article counts, they should be read as exploratory rather than definitive. Given recent macro-level evidence of strain in China–US scientific collaboration and talent mobility (Conroy, 2024), China’s comparatively limited international co-authorship may partly reflect structural conditions rather than field-specific choices; bibliometric maps cannot establish causality here, but the asymmetry suggests that broader multi-country partnerships could diversify both the questions addressed and the evidentiary base of this field.

A parallel redistribution is evident at the institutional level (**Table 2**; **Figure 3**), where the cumulative curves of the five most productive affiliations (**Figure 3A**)—which, like **Figure 2C**, aggregate all-author-affiliation output—display a clear chronological succession. St. Jude Children’s Research Hospital established the earliest sustained lead and Harvard University grew steadily in the early phase, consistent with the pioneering role of North American laboratories in influenza-based NLRP3 models (Allen et al., 2009; Thomas et al., 2009); after approximately 2018–2020, however, the Chinese institutions accelerated markedly. Wuhan University is a particularly suggestive case: it entered the field later than the other leading affiliations yet shows the steepest post-pandemic cumulative curve, reaching an output comparable to longer-established leaders. This trajectory may reflect local experience of the early COVID-19 outbreak, the concentration of respiratory-virus expertise in Wuhan, and rapid institutional mobilization, although our data cannot attribute the growth to any single factor. Conversely, the flattening of St. Jude’s curve after 2022 should be interpreted with caution: a change in slope reflects a change in annual output rather than a loss of accumulated leadership, and the plateau may therefore indicate slower annual growth, maturation, or reorientation of that institution’s research portfolio rather than withdrawal from the field. The presence of Nanjing University of Chinese Medicine among the ten most productive affiliations is consistent with the broad research attention devoted to traditional Chinese medicine in the fields of COVID-19 and other viral infections (Huang et al., 2021a). Taken together, the institutional and national curves converge on a coherent interpretation: the investment, public-health demand, laboratory expansion, and researcher growth that propelled China’s national output appear to have been realized through a set of high-output domestic institutions, exemplified by the Chinese Academy of Sciences’ steady rise to first place by 2025–2026. The institutional collaboration map (**Figure 3B**) qualifies this picture in the same way as the country network: Chinese institutions form densely connected domestic clusters, US institutions constitute a distinct North American cluster, and cross-cluster links, though visible, remain sparse. This suggests that the field’s fastest-growing segment is also its most self-contained and that multi-institutional, cross-national teams may be well positioned to connect these partly separate communities.

The journal landscape reveals a dual communication structure (**Figures 4**, **5**; **Tables 3**, **4**): although the WoSCC articles are dispersed across 521 journals, volume and influence are decoupled. High-volume specialist and open-access outlets, led by Frontiers in Immunology, sustain the field’s routine, incremental dissemination—consistent with cross-field evidence that open-access publishing has expanded the reach and visibility of rapidly growing literatures (Piwowar et al., 2018) —whereas citation impact is concentrated in a small number of landmark publications, most strikingly Nature, which accumulated 4,374 citations from only two articles (**Table 4**). The predominance of high-volume outlets among the most productive journals may also partly reflect changing journal-choice incentives under recent research-assessment reforms (Zhang and Sivertsen, 2020). These quantitative patterns describe attention and reach rather than research quality and, in line with the Leiden Manifesto, are best interpreted as supplements to, rather than substitutes for, qualitative expert judgment (Hicks et al., 2015). The co-citation network (**Figure 5**) integrates the two dimensions: Frontiers in Immunology, Nature, the Journal of Virology, and the Journal of Immunology emerge as central hubs, the latter two bridging the virology and immunology communities whose intersection defines this field. Together, the temporal, geographical, institutional, and journal-level patterns delineate how this field is organized and communicated.

### 4.2 Hotspots and development trends

As described above, the dual-database bibliometric analysis suggests that research frontiers and focal areas in this domain mainly concentrate on three themes: (1) diverse viral triggers and the upstream molecular cascades that activate NLRP3; (2) the tension between protective antiviral defense and pathological hyperinflammation; and (3) NLRP3-coupled regulated cell death and its therapeutic targeting. These three themes recur consistently across the highly cited literature (**Table 5**), citation-burst detection (**Figure 6**), and keyword co-occurrence clustering (**Figure 7**), and their relative prominence has shifted in an orderly chronological sequence (**Figure 8**), as detailed below.

#### 4.2.1 Three persistent core research hotspots

The first persistent hotspot concerns the diversity of viral triggers and the upstream mechanisms of NLRP3 activation. Citation-burst analysis (Results 3.4.2, **Figure 6**) shows that the three strongest bursts in the entire dataset all address this question: the report that influenza virus activates inflammasomes via its intracellular M2 ion channel (Ichinohe et al., 2010), the work of Allen et al. and Thomas et al. establishing NLRP3 as a mediator of *in vivo* innate defense against influenza A virus (Allen et al., 2009; Thomas et al., 2009), and the finding that the SARS-CoV-2 N protein promotes NLRP3 inflammasome activation to induce hyperinflammation (Pan et al., 2021). This bibliometric pattern indicates that virus-specific agonists—viroporins, viral nucleic acids, and structural proteins—have successively anchored the field’s mechanistic core, extending from influenza to coronaviruses (Rodrigues and Zamboni, 2025). Notably, the most highly cited papers in **Table 5** are predominantly broad conceptual frameworks, such as Toll-like receptor crosstalk, inflammaging, and pyroptosis/inflammatory caspase biology, rather than virus-specific experimental studies; it is therefore the burst literature, not the citation-volume literature, that identifies viral triggering as a central driver of the field. The keyword layer corroborates this structure: in the WoSCC co-occurrence map (Results 3.5.1, **Figure 7**), “activation” is the single most frequent term, cluster 4 gathers the canonical signaling vocabulary (inflammasome, IL-1β, receptor, recognition, replication), and cluster 2 links upstream sensing to RIG-I and Toll-like receptors; consistently, the canonical signaling vocabulary dominates the highest-frequency terms in **Table 6**. More recently, the burst literature suggests that this hotspot is consolidating toward a unified structural mechanism explaining how chemically unrelated viral products converge on the same sensor (**Figure 6**), a direction taken up in the third hotspot below.

The second hotspot captures the unresolved duality of NLRP3 as both a protective antiviral effector and a driver of pathological hyperinflammation. Keyword co-occurrence mapping (Results 3.5.1, **Figure 7**) separates these two faces into distinct but adjacent clusters: cluster 2 assembles the vocabulary of host defense—innate immunity, interferon, pattern-recognition receptors, vaccine, and adjuvant—whereas cluster 1 assembles the vocabulary of immunopathology, including COVID-19, acute lung injury, cytokine storm, neuroinflammation, fibrosis, and biomarker. Their coexistence within a single map mirrors the experimental evidence that moderate inflammasome activation may facilitate viral clearance, whereas sustained overactivation can aggravate tissue inflammatory damage (Sarvestani and McAuley, 2017). The citation-burst record reinforces the same tension from opposite directions: the influenza bursts positioned NLRP3 as a mediator of *in vivo* antiviral defense (Allen et al., 2009; Thomas et al., 2009), with the upstream viral trigger delineated in subsequent work (Ichinohe et al., 2010), while the recent SARS-CoV-2 bursts (Pan et al., 2021), together with patient-level evidence that inflammasome activation correlates with COVID-19 severity (strength 21.43) (Rodrigues et al., 2021), linked it to life-threatening hyperinflammation; experimental work further implicated TNF-α/IFN-γ-driven inflammatory cell death in severe SARS-CoV-2 infection and cytokine shock syndromes (Karki et al., 2021). **Table 6** quantifies how deeply this duality pervades the literature, with “inflammation” (n = 223), “innate immunity” (n = 158), and “COVID-19” (n = 165) all among the most frequent keywords. Critically, neither the bibliometric signal nor the underlying experimental literature resolves a quantitative or contextual threshold separating protective from pathological activation, and inconsistencies among *in vitro* systems, murine models, and human samples persist (Yue et al., 2024). This gap remains a central conceptual challenge, because the same pathway is invoked to justify both adjuvant strategies that enhance inflammasome-dependent defense and inhibitory strategies that suppress it.

The third hotspot links NLRP3 to regulated cell death and, through it, to therapeutic targeting. Cluster 3 of the keyword map (Results 3.5.1, **Figure 7**) organizes the cell-death vocabulary—pyroptosis, apoptosis, cell death, caspase-1, gasdermin D, necroptosis, and PANoptosis (Samir et al., 2020; Zheng and Kanneganti, 2020) —around the NLRP3-caspase-1-GSDMD axis (Miao et al., 2023), extending across pathogens such as respiratory syncytial virus and dengue virus and encompassing Z-DNA binding protein 1 (ZBP1)-driven cell death in influenza (Kuriakose et al., 2016); **Table 6** underscores the weight of this theme, with “pyroptosis,” “apoptosis,” and “cell death” among the most frequent keywords. The burst literature shows the same theme gaining recent momentum: the review on NLRP3 inflammasome activation and cell death (strength 16.47) (Huang et al., 2021b) and the structural analysis of inflammasome assembly (strength 14.11) (Fu and Wu, 2023) are among the most recent high-strength bursts in **Figure 6**, indicating that the field’s attention is shifting from describing cell-death phenotypes toward mechanistic and structural understanding. This structural turn matters translationally, because the druggable conformations and oligomeric interfaces defined by these assembly studies may convert the cell-death axis from a descriptive endpoint into a tractable target. Nevertheless, the bibliometric record itself underscores how early-stage this translation remains: in the integrated keyword analysis (Results 3.5.2), “inhibitor” occurred only 25 times, “drug efficacy” 54 times, and “drug mechanism” 70 times, and the prototypical NLRP3 inhibitor MCC950 (Coll et al., 2015) does not appear as a common keyword in either database. Early-phase agents such as GDC-2394 and DFV890 (Tang et al., 2023; Shen et al., 2025) exemplify a pipeline that has yet to establish clinical efficacy in viral respiratory disease. The third hotspot is therefore best characterized as mechanistically mature but therapeutically preclinical.

#### 4.2.2 Chronological evolution of research themes

The chronological dimension of these hotspots is defined here by the trend-topic analysis reported in Results 3.5.3 and visualized in **Figure 8**, which tracks the median occurrence period of author keywords across the WoSCC corpus. Read longitudinally, the map complements the static hotspot structure described above.

The trend-topic map resolves this evolution into four sequential phases. Between 2010 and 2014, trending terms comprised the field’s molecular parts list (e.g., cryopyrin/NALP3 and caspase-1 activation), reflecting a period in which even the sensor’s nomenclature had not yet standardized. From 2014 to 2018, the vocabulary shifted decisively toward viral recognition and host defense, providing bibliometric support for the influenza-centered era in which NLRP3 was established as an *in vivo* antiviral effector (Allen et al., 2009; Thomas et al., 2009). Between 2018 and 2022, attention consolidated around the NLRP3 inflammasome itself and NF-κB signaling within standardized mouse-model systems; during this phase the field deepened its mechanistic analyses across standardized experimental systems, and the pandemic-era surge in output (Results 3.1) was absorbed into this mechanistic mainstream. From 2022 to 2026, emerging terms—inhibition, therapy, regulator, NRF2, diagnosis, and brain—signal a translational turn accompanied by growing attention to brain-related pathology.

Taken together, the four sequential phases of the trend-topic map (**Figure 8**) describe a trajectory from component discovery, through viral recognition and host defense, to model-based signaling mechanisms, and finally toward therapeutic and diagnostic translation. Two interpretive cautions are warranted. First, trend topics record shifts in what authors write about, not in what has been validated; the prominence of “therapy” and “diagnosis” after 2022 reflects research attention rather than demonstrated clinical efficacy, a distinction developed in Section 4.3. Second, the phase boundaries are bibliometric medians rather than sharp transitions, and earlier themes—particularly inflammasome activation and cell death—persist beneath each successive layer, which is why the three hotspots in Section 4.2.1 remain simultaneously active.

### 4.3 Clinical and translational implications

Cross-database keyword analysis (Results 3.5.2) and the trend-topic trajectory (Results 3.5.3, **Figure 8**) permit a calibrated assessment of how close this field is to clinical application. The clearest signal is a pronounced translational imbalance. Human-oriented terms are frequent in absolute terms—”human” (n = 1,016), “human cell” (n = 380), and “adult” (n = 210)—yet explicitly clinical publications remain rare (“clinical article,” n = 76), whereas the experimental-model vocabulary is considerably larger, led by “animal experiment” (n = 616), “mice” (n = 611), and “animal model” (n = 551) (Results 3.5.2). The literature on NLRP3 in viral infection is therefore predominantly model-based, and conclusions drawn from murine knockout systems should not be extrapolated to patients without qualification (Yue et al., 2024).

The virus and disease spectrum contextualizes where translation is most advanced. As shown in Results 3.5.2, influenza and influenza A virus are the dominant virus-related terms, followed by COVID-19 (Scopus: n = 218; WoS: n = 165), HIV-1/HIV infections (n = 82), respiratory syncytial virus (n = 32), and smaller representation of hepatitis C virus (n = 15), dengue virus (n = 15), and Epstein-Barr virus (n = 13), with “coronavirus” (n = 26) bridging pre-pandemic and current literature (Results 3.5.2); experimental studies have linked several of these pathogens to NLRP3-associated mechanisms (Segovia et al., 2012; Negash et al., 2013; Wu et al., 2013; Rawat et al., 2019). Organ-level disease terms—acute lung injury, pneumonia, fibrosis, neuroinflammation, and viral myocarditis—map the clinical phenotypes toward which mechanistic work is oriented. This breadth implies that translational strategies will likely need to be virus- and organ-specific rather than generic.

A third implication concerns biomarkers, where keyword signals must be strictly distinguished from validated clinical evidence. Candidate markers are well represented bibliometrically (Results 3.5.2), but keyword frequency measures research attention, not diagnostic performance. Patient-level validation remains limited: the clearest verified example is the association between inflammasome activation in patient samples and COVID-19 severity (Rodrigues et al., 2021), and no standardized, pathogen-spanning cut-off values for IL-1β or IL-18 currently exist (Yue et al., 2024). These molecules should therefore be regarded as biomarker candidates pending prospective, multi-cohort validation.

Intervention-related terms are rising but remain modest in volume (Results 3.5.2), and the low frequency of inhibitor-related terms and the absence of MCC950 (Coll et al., 2015) as a common keyword are consistent with a predominantly preclinical landscape. Likewise, the post-2022 trend terms “therapy,” “inhibition,” “regulator,” “NRF2,” and “diagnosis” in **Figure 8** indicate rising attention rather than proven clinical benefit. A reasonable interpretation is that the field’s next phase should prioritize standardized human cohorts, virus-specific biomarker validation, and the movement of preclinical NLRP3-targeting strategies into rigorously designed clinical studies. Given the disproportionate burden of lethal respiratory viral–bacterial co-infection (Jamieson et al., 2013), establishing co-infection patient cohorts would further help convert bibliometric momentum into validated translational outcomes.

### 4.4 Limitations

This dual-database mapping has several inherent limitations, consistent with those reported for comparable NLRP3 bibliometric studies (Xiao et al., 2022; Chen et al., 2026).

1. Database and language bias. Only English-language original articles and reviews published before April 28, 2026 were included. Non-English publications and studies indexed only in regional databases may be underrepresented, which may bias estimates of global output and collaboration patterns. WoSCC and Scopus adopt inconsistent metadata frameworks; we therefore analyzed the datasets separately to avoid information loss.
2. Citation metrics cannot fully represent research quality. High citation burst strength reflects research attention and visibility rather than experimental rigor, and many highly cited manuscripts contain contradictory *in vitro* and *in vivo* results (Yue et al., 2024). This study performed quantitative citation statistics without a systematic meta-analysis to reconcile conflicting mechanistic findings across different viral models.
3. Grey literature exclusion. Preprints, conference abstracts, and unpublished clinical trial data were excluded, as is standard in bibliometric workflows; very recent findings may therefore be underrepresented. The data collection cutoff date was April 28, 2026, so complete full-year 2026 publications were not captured.
4. Lack of author disambiguation analysis. Author collaboration networks were not constructed, and homonymous author names cannot be fully distinguished, which may cause minor deviations in institutional output statistics; future studies could address this with author-disambiguation methods.

Despite these limitations, this study provides, to our knowledge, the first dual-database bibliometric analysis of NLRP3 inflammasome research across all viral infectious diseases. The visualized publication trends and cross-institutional cooperation networks may offer useful orientation for virologists, innate immunologists, and researchers developing NLRP3-targeted strategies.

## 5 Conclusion

This study presents a dual-database bibliometric analysis of NLRP3 inflammasome research in viral infections, integrating 1,544 publications from the WoSCC and 1,495 records from Scopus to map the global research landscape and identify dominant trends, collaborative networks, and translational frontiers. The principal findings are summarized as follows:

- Annual output evolved through distinguishable phases—foundational (2009–2013), steady expansion (2014–2019), pandemic-era surge (2019–2022), and post-2022 moderation and rebound—peaking at 213 WoSCC articles in 2025; the 2026 count reflects an incomplete publication year.
- The field shows dual leadership by China and the United States, with China overtaking the United States in cumulative all-affiliation output around 2021–2022 while remaining predominantly domestically co-authored. Whether this asymmetry represents untapped collaborative potential remains an open question.
- Three enduring research hotspots have shaped the intellectual trajectory of this domain: (1) diverse viral upstream agonists and conserved or virus-specific NLRP3 activation cascades; (2) the dual protective and pathological regulatory functions of NLRP3 inflammasome signaling; and (3) the NLRP3-pyroptosis axis intersecting with PANoptosis, for which therapeutic translation remains at a predominantly preclinical stage.
- Trend-topic analysis (**Figure 8**) suggests that, after 2022, research attention has shifted toward therapeutic targeting, regulatory pathways such as NRF2, diagnosis, and brain-related pathology. These signals represent research attention rather than validated clinical benefit.
- Future priorities suggested by these bibliometric frontiers include prospective, multi-cohort validation of candidate biomarkers such as IL-1β and IL-18; rigorously designed clinical studies of NLRP3-targeting strategies in severe viral respiratory disease; and the establishment of co-infection patient cohorts to inform combined antiviral and anti-inflammatory treatment strategies.

This analysis has several methodological limitations, including restriction to English-language publications, reliance on citation metrics that cannot fully capture research quality, exclusion of grey literature, incomplete capture of the 2026 publication year, and the absence of formal author-disambiguation analysis.

In summary, this mapping provides an orientation framework for virologists, innate immunologists, and translational drug developers working on NLRP3-targeted strategies for viral infectious diseases. Bibliometric maps indicate attention, structure, and trends rather than mechanistic or clinical proof; converting the observed momentum into validated clinical outcomes will require the prospective experimental and clinical work outlined above.

## Data Availability

The original contributions presented in the study are included in the article/**Supplementary Material**. Further inquiries can be directed to the corresponding authors.
