# Supplementary material for: Advances in NLRP3 inflammasome research in viral infections: evolution, trends, and future directions
Source: Front Cell Infect Microbiol. 2026 Sep 10;16:1956027. doi: 10.3389/fcimb.2026.1956027 (PMC13600971; doi:10.3389/fcimb.2026.1956027)
Supplement: Supplementary file 1 [file Table1.docx]

**Web of Science database search formula**

((TS=("Viral Infection*" OR "Viral Disease*" OR virus OR viruses) AND TS=("NLR family pyrin domain containing 3 protein" OR NLRP3* OR "cold autoinflammatory syndrome 1 protein" OR "nod-like receptor protein 3 inflammasome" OR "NACHT, LRR and PYD domains containing protein 3")) AND DT=(Article OR Review)) AND LA=(English)

Results: 1544

**Scopus database search formula**

TITLE-ABS-KEY ( "Viral Infection*" OR "Viral Disease*" OR virus OR viruses ) AND TITLE-ABS-KEY ( "NLR family pyrin domain containing 3 protein" OR NLRP3* OR "cold autoinflammatory syndrome 1 protein" OR "nod-like receptor protein 3 inflammasome" OR "NACHT, LRR and PYD domains containing protein 3" ) AND ( LIMIT-TO ( DOCTYPE , "ar" ) OR LIMIT-TO ( DOCTYPE , "re" ) ) AND ( LIMIT-TO ( LANGUAGE , "English" ) )

Results: 1495
