# Supplementary material for: Advances in NLRP3 inflammasome research in viral infections: evolution, trends, and future directions
Source: Front Cell Infect Microbiol. 2026 Sep 10;16:1956027. doi: 10.3389/fcimb.2026.1956027 (PMC13600971; doi:10.3389/fcimb.2026.1956027)
Supplement: Supplementary file 2 [file Table2.docx]

Vosviewer parameters (**WoSCC database**)

In the co-authorship network analysis, the following thresholds were established: a minimum of 5 documents per country and a minimum of 8 documents per institution. For the co-citation analysis of sources, the minimum citation count for a journal was set at 115. In the keyword co-occurrence analysis, the minimum frequency of occurrence for a keyword was established at 12. Additionally, general terms such as “viral infection,” “virus,” “nlrp3,” “nlrp3 inflammasome,” and “nlrp3 inflammasome activation” were excluded, and synonymous keywords were consolidated to enhance the clarity of the mapping.

Vosviewer parameters (**Scopus database**)

For the co-occurrence keyword analysis, we required a minimum of 52 occurrences for each keyword, and excluded terms used in the search query as well as their synonyms.
